# Chemically defined human vascular laminins for biologically relevant culture of hiPSC-derived brain microvascular endothelial cells

**DOI:** 10.1186/s12987-020-00215-2

**Published:** 2020-09-10

**Authors:** Pedram Motallebnejad, Samira M. Azarin

**Affiliations:** Department of Chemical Engineering and Materials Science, University of Minnesota, Minneapolis, MN 55455 USA

**Keywords:** Laminin 511, Brain specific microvascular endothelial cells, Shear stress, Human induced pluripotent stem cells, Basement membrane

## Abstract

**Background:**

In recent years, differentiation of human induced pluripotent stem cells (hiPSCs) into brain-specific microvascular endothelial cells (iBMECs) has frequently been used to model the blood–brain barrier (BBB). However, there are limitations in the use of iBMECs for in vitro studies, such as transendothelial electrical resistance (TEER) instability, weak junctional expression of VE-cadherin, and lack of proper fluid shear stress response. In vivo, the basement membrane (BM) composition of the BBB evolves throughout development, and laminins become the dominant component of the mature vascular BM. However, laminin isoforms of the endothelial BM have not been used for culture of differentiated iBMECs. The main goal of this study is to investigate the effect of different laminin isoforms of the endothelial BM on iBMEC functionality and to determine whether better recapitulation of the physiological BM in vitro can address the aforementioned limitations of iBMECs.

**Methods:**

Using a previously reported method, hiPSCs were differentiated into iBMECs. The influence of main laminins of the endothelial BM, LN 411 and LN 511, on iBMEC functionality was studied and compared to a collagen IV and fibronectin mixture (CN IV-FN). Quantitative RT-PCR, immunocytochemistry, and TEER measurement were utilized to assess gene and protein expression and barrier properties of iBMECs on different extracellular matrices. Single-channel microfluidic devices were used to study the effect of shear stress on iBMECs.

**Results:**

LN 511, but not LN 411, improved iBMEC barrier properties and resulted in more sustained TEER stability. Immunocytochemistry showed improved junctional protein expression compared to iBMECs cultured on CN IV-FN. iBMECs cultured on LN 511 showed a reduction of stress fibers, indicating resting endothelial phenotype, whereas gene expression analysis revealed upregulation of multiple genes involved in endothelial activation in iBMECs on CN IV-FN. Finally, culturing iBMECs on LN 511 enhanced physiological responses to shear stress, including morphological changes and enhanced junctional protein association.

**Conclusion:**

LN 511 improves the functionality and long-term barrier stability of iBMECs. Our findings suggest that incorporation of physiologically relevant LN 511 in iBMEC culture would be beneficial for disease modeling applications and BBB-on-a-chip platforms that accommodate fluid flow.

## Background

Blood brain-barrier (BBB) is a term used to describe the microvasculature of the brain, which is composed of brain-specific microvascular endothelial cells (BMECs) and possesses unique properties including a highly tight paracellular barrier that limits the free diffusion of substances into and out of the brain and expression of specific ion channels, efflux transporters, and receptors that selectively transfer the required ions and molecules across the BBB [1]. Human induced pluripotent stem cell (hiPSC)-derived BMECs (iBMECs) have recently been developed to model the BBB in vitro [2, 3]. iBMECs possess superior barrier properties compared to primary BMECs and existing BMEC cell lines, and they are invaluable for genetic disease modeling and personalized medicine applications [2]. However, some reports have shown that the barrier properties of these cells are not stable when the cells remain in culture for an extended period of time [4–6]. In addition, expression of VE-cadherin, an essential endothelial marker, is not distinctly junctional in some reports [6–8]. There is also concern about the developmental stage and maturation of stem cell-derived cells, including iBMECs [9]. Shear stress acting on endothelial cells due to the presence of flow is a significant factor that contributes to endothelial differentiation and function [10–13]. Applying fluid shear stress to primary BMECs results in upregulation of essential adherens and tight junction proteins and a significant increase in expression of cytoskeletal proteins as well as inhibition of endothelial proliferation [14]. However, studies using iBMECs point out the lack of response to shear stress in terms of junctional protein expression enhancement and morphological changes [15]. Identifying and incorporating key factors of the in vivo BMEC microenvironment into the in vitro development and maintenance of iBMECs could address these current limitations.

In the original iBMEC differentiation protocol and most of the subsequent studies using iBMECs, type IV collagen and fibronectin have been utilized to provide the iBMECs with an adhesion layer. Fibronectin, a commonly used cell adhesion molecule, plays a major role during development and some pathological conditions; however, it is not in contact with endothelial cells of healthy adults [16, 17]. In vivo, the basement membrane (BM) underneath epithelial and endothelial cells that separates them from connective tissue [18] consists of two major networks of proteins, laminin and type IV collagen. Laminins, of which there are at least 16 isoforms that exhibit tissue and developmental stage specificity [19], are major players in physiological endothelial-BM interactions. While the laminin network closely interacts with the receptors on the cell surface and is the only required component for BM assembly and formation [20], the type IV collagen network stabilizes the BM and protects it from mechanical stress [21, 22]. Interestingly, the BM composition of the BBB and the expression of integrins on the BMECs change during development. In vivo studies have shown that BMEC integrin expression switches from α4β1 and α5β1 to α1β1 and α6β1 during maturation of the brain vasculature [23]. Furthermore, the expression of fibronectin, which binds to α4β1 and α5β1 integrins, is downregulated, while the expression of laminins, which interact with α6β1 integrin, is upregulated during development [23]. These results suggest that there is a switch from fibronectin-mediated signaling during angiogenesis in development to laminin-mediated signaling during maturation. In addition, laminins are involved in many physiological phenomena concerning blood vasculature such as shear stress response, cancer and immune cell extravasation, and endothelial junctional tightness [16, 24, 25]. Accordingly, culture of iBMECs on laminins may be more suitable for maintaining physiologically relevant functionality.

Recently, the use of laminin isoforms during the differentiation of iBMECs has been studied, suggesting laminin 221 (LN 221) as an enhancer of iBMEC barrier properties, but this study did not evaluate the effect of laminin isoforms of the endothelial BM on the culture of differentiated iBMECs [26]. Another study reported the effect of different ECM proteins including laminin on TEER of differentiated iBMECs; however, specific laminin isoforms of the endothelial BM were not investigated [27]. Laminin 411 (LN 411) and laminin 511(LN 511) are the two main isoforms that are present in the endothelial BM of mature vasculature [17, 24, 28–30]. In contrast to LN 411, which is detectable in the BM of all blood vessels throughout development, LN 511 appears in the endothelial BM of some blood vessels after birth, roughly at the time of pericyte recruitment to the developing vessels [17]. Pericytes, together with endothelial cells, contribute to the formation of mature endothelial BM by secreting LN 411 and LN 511 [17, 31–33]. In addition, interaction of endothelial cells and pericytes results in alteration of BM protein secretion profile in both cell types [34]. The brain possesses the highest density of pericytes, and inhibition of laminin production by pericytes in conditional knockout mice results in BBB breakdown in an age dependent manner, suggesting a key role of pericytes in vascular BM formation in the brain [35]. In mature vasculature, the LN 411 isoform is continuously expressed throughout the vascular tree independent of vessel type; however, expression of LN 511 is nonuniform and patchy in venules and postcapillary venules and absent in the BM of some fenestrated capillaries and peritubular capillaries in the kidney [31]. Capillaries and arterioles of most other organs and tissues, including the BBB, exhibit uniform expression of LN 511 in their BM [33]. Previous in vitro studies suggest that LN 511, but not LN 411, increases the tightness of the endothelial barrier in primary rat brain endothelial cells and the bEND.5 cell line [16, 36]. LN 411 and LN 511 interact with distinct sets of integrins and exhibit substantially different integrin-binding affinity, with LN 511 binding to more integrins with stronger binding affinity [37, 38]. As a result, endothelial cell interactions with each of these laminin isoforms can activate distinct signaling pathways and cellular responses.

Due to difficulties in isolation and purification of specific isoforms of native pure laminins from tissues, the use of tissue-specific laminins for in vitro studies has been limited [39]. The laminins that have traditionally been commercially available are laminin 111 (from Engelbreth-Holm-Swarm murine sarcoma basement membrane), a mixture of laminin 211 and laminin 221 (from human placenta), or a mixture of various laminin isoforms (pepsinized human laminin), which are not specific to the BM of endothelial cells. Recently, recombinant laminins and laminin fragments have been produced, enabling the use of specific isoforms of laminins for in vitro studies [20, 39–41]. In addition to being more physiologically relevant molecules for modeling the BM in vitro, recombinant laminins are chemically defined and do not suffer from limitations of animal-derived extracellular matrix (ECM) molecules such as batch-to-batch variability [42].

BM molecules are known to control the function of endothelial cells through interaction with cellular integrins, and they contribute to inducing an activated endothelial phenotype [43]. For instance, endothelial laminins and laminin-binding α6β1 are the predominant BM component and integrin, respectively, in normal mature vessels of the central nervous system; however, expression of fibronectin and fibronectin-binding α5β1 rapidly increases when angiogenesis begins after hypoxia [44–46], suggesting a key role of integrin-BM interactions in endothelial activation. Activation of endothelial cells happens in different pathological conditions such as inflammation and angiogenesis and results in various cellular responses, including stress fiber formation, increased permeability, and changes in gene and protein expression [47, 48]. Binding of integrins to different ECM molecules regulates the organization of the actin cytoskeleton and actin filament assembly [49]. Generally, two types of actin bundles are observed in endothelial cells: junction-associated circumferential actin filaments, which are mainly found in mature, resting vasculature, and contractile actomyosin bundles, also known as stress fibers, which are observed in activated endothelial cells during various physiological and pathological conditions [50]. One consequence of stress fiber formation is elevation of endothelial layer permeability due to the formation of gaps in intercellular regions [47]. Furthermore, matrix metalloproteinases are upregulated in inflammation and angiogenesis and are involved in remodeling of BM and degradation of tight junctions, thereby increasing the endothelial layer permeability [51, 52]. Angiopoietin-2 (Ang-2), an autocrine molecule that destabilizes the endothelial layer [53], is also involved in angiogenesis and inflammation and its expression is upregulated in various diseases [54]. Due to appearance of stress fiber formation together with upregulation of MMPs and Ang-2 in various BBB pathologies that result in endothelial activation, these markers are commonly analyzed to identify the activated endothelium. Since fibronectin is upregulated in many vascular pathologies that result in endothelial activation, while laminins are the main component of healthy adult vascular BM, the use of endothelial BM laminins instead of fibronectin could help maintain endothelial cells in a resting, non-activated phenotype.

In this study, we examined the effect of endothelial BM laminins, LN 411 and LN 511, on iBMEC functionality in comparison to the standard mixture of collagen IV and fibronectin (CN IV-FN). E8 fragments of LN 511 and LN 411 (LN 511-E8 and LN 411-E8, respectively), which retain most of the integrin binding activity of full length laminins and are commonly used as an alternative to full length laminins for in vitro studies [39, 55–58], were used for the majority of the experiments in this work. TEER measurement, immunocytochemistry, and qRT-PCR were utilized to investigate changes in iBMEC barrier properties, junctional protein expression, and alteration of genes associated with activated endothelial phenotype. Our results indicate that iBMECs exhibit a resting endothelial phenotype with improved long-term barrier stability when cultured on LN 511-E8 compared to the standard CN IV-FN mixture. In addition, we show that culturing iBMECs on LN 511-E8 improves their shear stress response, as indicated by changes in cell morphology, surface area, and junctional protein expression. As these results show that the BM has a significant influence on iBMEC functionality, we think the use of the physiologically relevant and chemically defined LN 511-E8 molecule is essential for studies that require a healthy BBB phenotype for an extended time, such as mechanistic studies of various damage-inducing factors on the BBB, neurological disease modeling and investigating the effect of potential therapeutics on the BBB, and analyzing cancer or immune cell interactions with the BBB.

## Methods

### Cell culture and differentiation

iBMECs were differentiated from hiPSCs (IMR-90-4 cells from WiCell and ACS-1024 from ATCC) according to Stebbins et al. [59]. On day 8, cells were placed onto CN IV-FN coated plates for selective purification of endothelial cells. After 1 to 2 h, unattached cells were removed, and cells were washed once with Dulbecco’s Phosphate-Buffered Saline (DPBS). The purified endothelial cells were then subcultured at a density of 500,000 cells/cm^2^ onto ECM protein-coated ThinCert cell culture inserts (Greiner Bio-One), well plates or ibidi μ-slides and cultured for 1 day to reach 100% confluency in endothelial cell medium, which consists of human endothelial serum free medium (Thermo Fisher Scientific) supplemented with 1% bovine platelet-poor plasma derived serum (Alfa Aesar) or 2% fetal bovine serum (Thermo Fisher Scientific), 20 ng/ml basic fibroblast growth factor (bFGF) (PeproTech), and 10 μM retinoic acid (Millipore Sigma). After approximately 24 h, the medium was changed to endothelial cell medium without retinoic acid and bFGF. The medium was not changed after this point. iBMECs that were subcultured into well-plates were used for qRT-PCR, while the cells in ibidi μ-slides and ThinCert cell culture inserts were used for immunocytochemistry. The well plates, ThinCert cell culture inserts, and ibidi μ-slides were pre-coated with the ECM proteins: 1 μg/cm^2^ LN 511-E8 or LN 411-E8 (iMatrix, iWAi), 1 μg/cm^2^ full length laminin 511 (Biolamina), 100 μg/ml fibronectin (Millipore Sigma), or a mixture of 400 μg/ml collagen IV (Millipore Sigma) and 100 μg/ml of fibronectin.

### TEER measurements

TEER was evaluated using the EVOM2 voltohmmeter with STX2 chopstick electrodes (World Precision Instruments). TEER was measured 1 day after iBMECs were subcultured onto cell culture inserts (ThinCerts) and approximately every 24 h thereafter. A ThinCert coated with the ECM proteins, but without cells, was used to subtract the medium and membrane effects on TEER. The reported values are multiplication of surface area and measured electrical resistance of the iBMEC layer to eliminate the effect of cell culture area on the electrical resistance.

### Immunocytochemistry and F-actin staining of iBMECs

The primary antibodies and the secondary antibodies used in this study and their dilution factors are listed in Additional file 1: Tables S1 and S2. iBMECs cultured on ibidi μ-slides or cell culture inserts were fixed for 15 min in 4% paraformaldehyde (PFA) and permeabilized with 0.3% Triton X-100 in DPBS for another 10 min. Subsequently, cells were blocked for 1 h in 10% goat serum at room temperature. Next, cells were incubated overnight at 4 °C or for 1 h at room temperature with primary antibodies diluted in 10% goat serum in DPBS. After aspirating primary antibody solutions, cells were washed three times with DPBS prior to adding 1:200 diluted secondary antibodies in 10% goat serum solution. Samples were washed three times with DPBS and stained with DAPI. For F-actin staining, after blocking the samples, cells were incubated for 30 min with 0.165 μM Alexa Fluor 488-conjugated phalloidin (Thermo Fisher Scientific) at room temperature and then washed three times with DPBS. Fluorescence imaging in Figs. 2, 3, 4, and Additional file 1: Figures S2 and S5 was performed on a Nikon TiE stand with an A1Rsi confocal scan head, powered by NIS-Elements confocal software (Nikon, Japan). A 20× objective with numerical aperture of 0.75 (Figs. 2, 3, and Additional file 1: Figures S2 and S5) or a 60× objective with numerical aperture of 1.4 (Fig. 4d–i) were used for confocal imaging. Excitation for fluorescence images was performed sequentially via 405, 488, and 561 nm lasers. Laser intensity and exposure were identical for imaging of a specific protein in different conditions within each figure, and NIS-Elements software was used for image visualization. Fluorescence images in Fig. 6 and Additional file 1: Figures S4 and S8 were obtained using the EVOS FL Auto microscope (Thermo Fisher Scientific).

### Fluid endocytosis assay

Four days after subculture onto LN 511-E8 or CN IV-FN coated ibidi μ-slides, rhodamine B-labeled 10 kDa neutral dextran (ThermoFisher Scientific) was added to the iBMEC culture medium at a concentration of 1 mg/ml and incubated for 50 min. Next, iBMECs were washed 3 times with ice cold PBS and fixed in 4% PFA for 15 min. After fixation, iBMECs were stained with wheat germ agglutinin (WGA)-Oregon Green 488 conjugate (ThermoFisher Scientific) for 5 min. iBMECs were washed 3 times with PBS and imaged using a Nikon TiE stand with an A1Rsi confocal scan head, powered by NIS-Elements confocal software via a 60× objective with numerical aperture of 1.4. Two images were taken from the middle of each well, and 4 independent wells were imaged per condition for each biological replicate. Images were analyzed in Fiji (ImageJ) to quantify the number of vesicles and pixel intensity. The macro code used to analyze the images can be found in Additional file 1.

### Gene expression analysis

RNA was extracted using the RNeasy Mini Kit (Qiagen) following the manufacturer’s instructions. QIAshredder columns (Qiagen) were used to homogenize the cell lysate, and the RNase-free DNase Set (Qiagen) was used to remove any residual DNA. Using an OmniScript Reverse Transcriptase Kit (Qiagen) and Oligo(dT)20 Primers (Life Technologies), RNA was reverse-transcribed into cDNA. Quantitative PCR was conducted using PrimePCR SYBR Green Assays (Bio-Rad) and iTaq Universal SYBR Green Supermix (Bio-Rad). Primers used in this study are listed in Additional file 1: Table S3. Relative expression of mRNA was quantified using the cycle threshold (C_t_) determined by the BioRad CFX Connect Real-Time PCR Detection System and the ΔΔC_t_ method. GAPDH was used as the housekeeping gene in the qRT-PCR experiments.

### Wound healing assay

Differentiated iBMECs were purified on CN IV-FN and subsequently subcultured onto 2-well plates with silicone inserts (ibidi) designed for the wound healing assay. The wells were pre-coated with LN 511-E8 or CN IV-FN. The next day, the inserts were removed and the medium was changed to endothelial cell medium without bFGF and retinoic acid. Using an EVOS FL Auto microscope, phase contrast images were taken every 3 h from the same spot, and the area void of cells in each image was calculated using ImageJ. Three independent replicates per condition were evaluated for this experiment.

### Evaluating the effect of shear stress on iBMECs

iBMECs were seeded in microfluidic devices with a single rectangular channel fabricated by soft lithography, where the channel width and height were 800 μm and 100 μm, respectively. The channels were pre-coated with LN 511-E8 or CN IV-FN, and purified iBMECs were seeded into the pre-coated devices and maintained under static conditions for 1 day. The next day, half of the samples were connected to a syringe pump and were cultured under dynamic conditions for 24 h. The shear stress value acting on the iBMECs was 4 dyne/cm^2^. This is in the range of physiological flow for larger capillaries and close to the shear stress that induces responses in primary BMECs [14, 60]. iBMECs in the device were fixed with 4% PFA and prepared for immunostaining and microscopy.

iBMECs stained for ZO-1 were used for cellular shape analysis due to clarity and distinction of the borders between the adjacent cells and negligible background signal. The ZO-1 images were converted to binary images and using the Shape Descriptor tool in Fiji (ImageJ), the average surface area, aspect ratio, and circularity of the cells were analyzed. The macro code used to analyze the images in Fiji can be found in Additional file 1. The inverse of aspect ratio ($\frac{minor\,axis}{major \,axis}$), a measure of elongation, and circularity ($\frac{4 \times Area}{{\left( {Perimeter} \right)^{2} }}$) are reported in this study. For reference, the circularity and inverse of aspect ratio of circles are 1, and these values become smaller for more elongated cells. Circularity also strongly depends on angularity of the analyzed shape. Therefore, inverse of aspect ratio is more suitable for measuring elongation.

### Statistical analysis

Cell shape analysis data are reported by the median with interquartile range. All the other data are presented as the mean ± standard deviation. Unless otherwise stated in the figure legend, an unpaired Student’s t test was used to calculate the P values for data with less than 5 samples, and a Mann–Whitney test was performed for cell shape analysis results due to non-normality of data as determined by the Kolmogorov–Smirnov test. P values smaller than 0.05 were reported as statistically significant. Further information on P values can be found in the figure legends. Reported results are representative of three independent differentiations (biological replicates) each with three technical replicates per condition unless otherwise noted in the figure legend.

## Results

### LN 511 and LN 511-E8 improve long-term iBMEC TEER stability

**Fig. 1:**
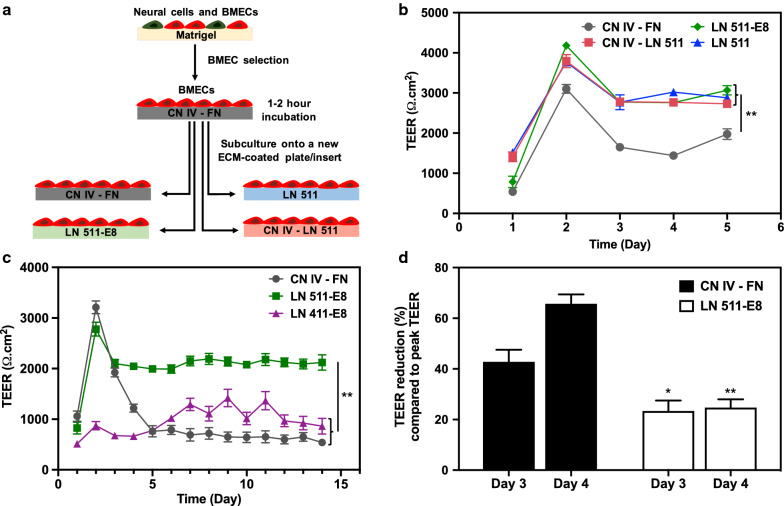
TEER measurement results for iBMECs differentiated from IMR-90-4 hiPSCs after subculture on different ECM proteins. **a** Schematic of iBMEC purification and cell seeding on different protein-coated surfaces. **b** TEER results for the purified iBMECs subcultured on different ECM protein-coated inserts for 5 days. **indicates P < 0.001 compared to CN IV-FN group. Differences between other groups are not statistically significant. **c** TEER measurement to evaluate long-term stability of the barrier after subculture onto LN 511-E8, LN 411-E8, or CN IV-FN. **indicates P < 0.001 compared to CN IV-FN group. Differences between LN 411-E8 and CN IV-FN samples are not statistically significant. A two-factor repeated measures ANOVA was used to analyze TEER data in (**b**) and (**c**). **d** Percent reduction in TEER compared to the peak TEER (TEER value on day 2) for the results shown in (**c**), indicating formation of a more stable barrier for iBMECs cultured on LN 511-E8. * indicates P < 0.05 and ** indicates P < 0.001 compared to CN IV-FN samples on the same day

Differentiated cells were incubated on CN IV-FN for 1–2 h in order to selectively purify the iBMECs. Purified iBMECs were then seeded on cell culture inserts coated with CN IV-FN, CN IV-LN 511, LN 511, or LN 511-E8 (Fig. 1a). One day after seeding the cells, iBMECs that were cultured on CN IV-LN 511 and LN 511 exhibited higher TEER than the other two conditions (Fig. 1b); however, iBMECs on LN 511-E8 showed the highest TEER on day 2, which is considered to be the peak TEER day. The TEER value remained significantly higher for CN IV-LN 511, LN 511, and LN 511-E8 samples compared to CN IV-FN samples for at least 5 days (Fig. 1b). While repeating the experiments showed the peak TEER might not always be higher for LN 511-E8 coated surfaces (Fig. 1c), the reduction in TEER on day 3 and day 4 was consistently lower for the cells on LN 511-E8 compared to CN IV-FN (Fig. 1d). Additionally, measuring TEER values for 2 weeks demonstrated that the TEER remains high, and there is negligible fluctuation in TEER for LN 511-E8 samples (Fig. 1c), making it suitable for long-term in vitro studies. LN 411-E8 was also used for TEER measurement experiments, but in some cases iBMECs failed to establish a monolayer barrier. An example of TEER values for iBMECs on LN 411-E8, when a barrier was formed, is shown in Fig. 1c. Starting TEER values for iBMECs on LN 411-E8 were significantly lower than both the LN 511-E8 and CN IV-FN coated samples. Moreover, there was significant fluctuation in recorded TEER on LN 411-E8 (Fig. 1c). Overall, iBMECs on LN 511 and LN 511-E8 demonstrated stable and high TEER values, especially after the peak TEER day. Due to the similar performance of full length LN 511 and LN 511-E8, LN 511-E8 was primarily used for the remaining experiments. To demonstrate the robustness of the approach, the efficacy of LN 511-E8 was also confirmed using iBMECs differentiated from the ACS-1024 cell line (see Additional file 1: Figure S1).

### Stronger junctional expression of endothelial adherens junction proteins is achieved on LN 511-E8

**Fig. 2:**
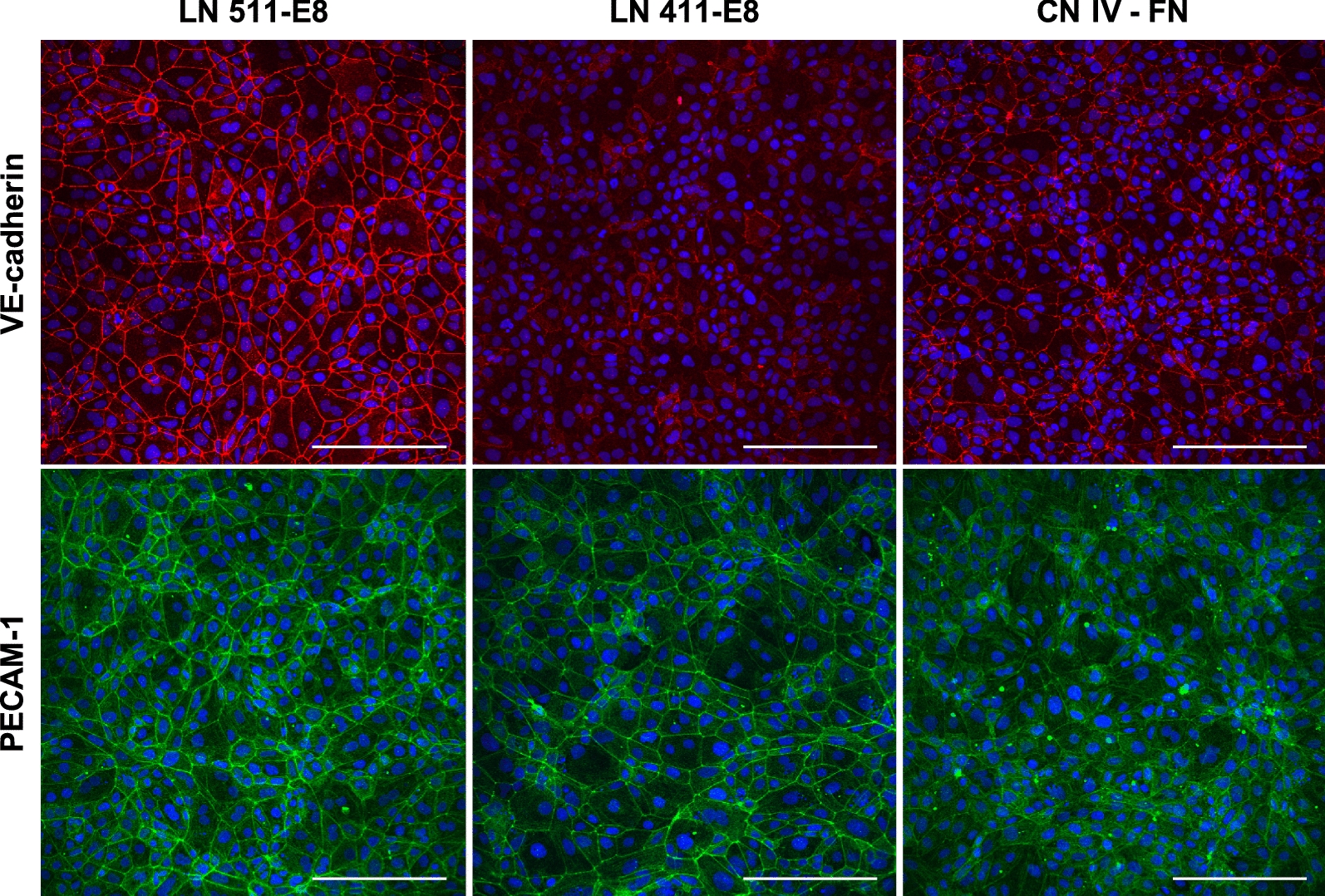
Immunocytochemistry of adherens junction proteins 2 days after subculture (peak TEER day). Fluorescence images of VE-cadherin (top row; red) and PECAM-1 (bottom row; green) in iBMECs cultured on LN 511-E8, LN 411-E8, or CN IV-FN. Nuclei are stained with DAPI and indicated in blue. Images are maximum intensity projections of confocal z-stacks. Scale bars indicate 200 μm

**Fig. 3:**
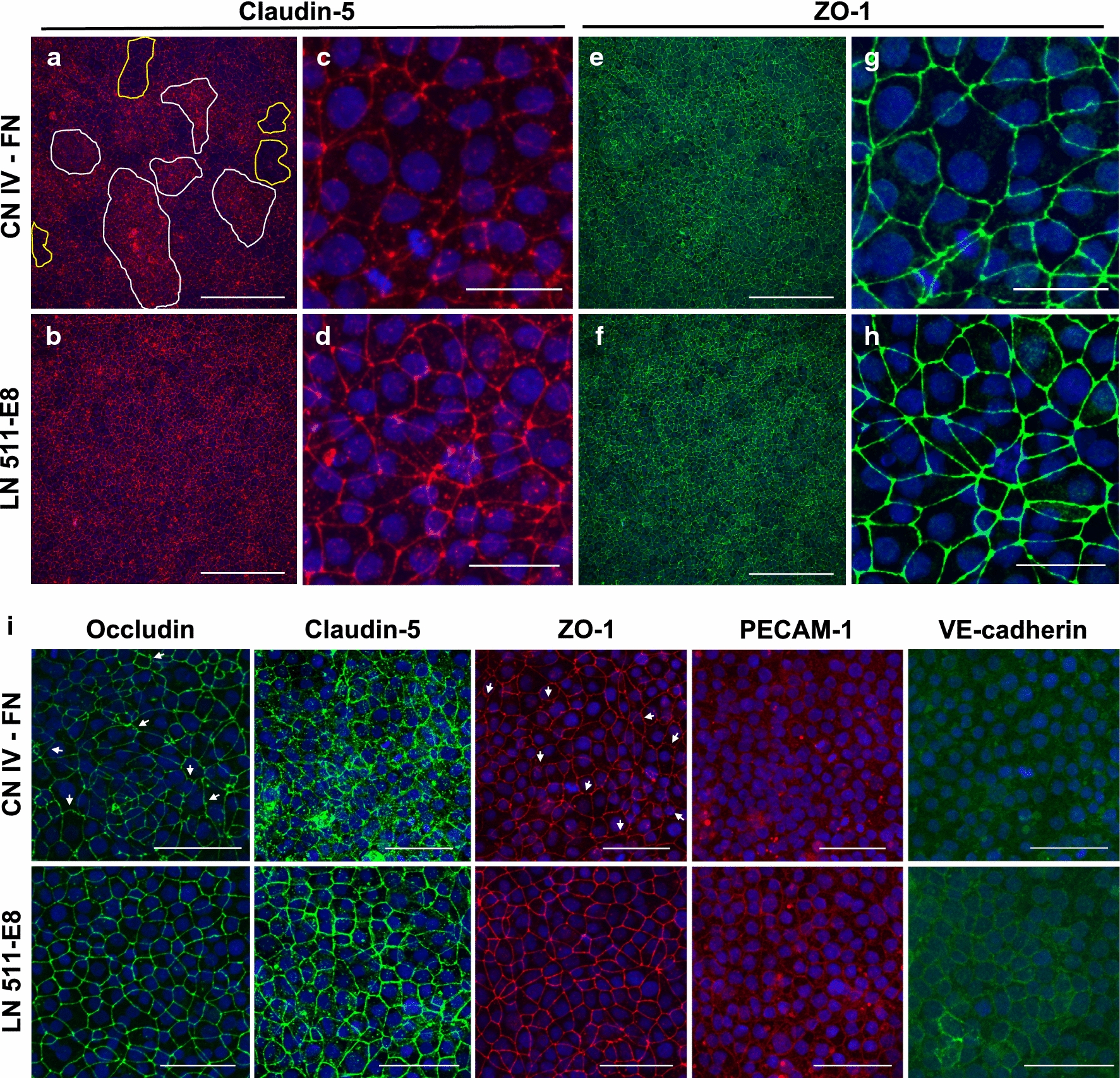
Immunocytochemical analysis of tight junction and adherens junction proteins 4 days after subculture. **a**–**h** Fluorescence images of iBMECs cultured in Thincert inserts. **a**, **b** Low magnification images of claudin-5 staining on CN IV-FN (**a**) and LN 511-E8 (**b**). Scale bars indicate 200 μm. In (**a**), examples of regions with internalized claudin-5 are outlined in white, while regions with low junctional expression of claudin-5 are outlined in yellow. **c**, **d** Higher magnification images of claudin-5 staining on CN IV-FN (**c**) and LN 511-E8 (**d**). Scale bars indicate 25 μm. The images in (**c**) and (**d**) are from regions with low claudin-5 expression. **e**, **f** Low magnification images of ZO-1 staining on CN IV-FN (**e**) and LN 511-E8 (**f**). Scale bars indicate 200 μm. **g**, **h** Higher magnification images of ZO-1 staining on CN IV-FN (**g**) and LN 511-E8 (**h**). Scale bars indicate 25 μm. Note that for (**a**–**h**) the samples are double-stained, so claudin-5 and ZO-1 images are shown for the same cells in each group. **i** Immunostaining of junctional proteins for iBMECs cultured on ibidi μ-slides coated with CN IV-FN (top row) or LN 511-E8 (bottom row). Claudin-5 staining on CN IV-FN shows a region with significantly internalized claudin-5. White arrows on occludin and ZO-1 images indicate examples of frayed or jagged junctions. Scale bars indicate 50 μm. All images in (**a**–**i**) are maximum intensity projections of confocal z-stacks

VE-cadherin is an endothelial-specific adherens junction molecule that contributes to various endothelial processes such as junctional integrity, shear stress response, proliferation, and apoptosis [61, 62]. After 2 days of subculture, immunostaining of iBMECs seeded on fibronectin-coated surfaces showed undetectable junctional expression of VE-cadherin (see Additional file 1: Figure S2). On the other hand, iBMECs on LN 511-E8 exhibited strong junctional VE-cadherin expression, while iBMECs on CN IV-FN had detectable junctional VE-cadherin expression, but with weaker intensity than on LN 511-E8 (Fig. 2 and see Additional file 1: Figure S2). Junctional expression of VE-cadherin was patchy in iBMECs cultured on LN 411-E8 coated surfaces (Fig. 2). Further, junctional expression of PECAM-1 was stronger on LN 511-E8 and LN 411-E8 compared to CN IV-FN (Fig. 2). Four days after subculture, junctional expression of both VE-cadherin and PECAM-1 was still stronger on LN 511-E8 compared to CN IV-FN; however, compared to day 2, junctional expression of both proteins decreased (Fig. 3i). Additionally, qRT-PCR analysis showed upregulation of VE-cadherin (*CDH5*) gene expression in some independent biological replicates while its expression remained unchanged in others (see Additional file 1: Figure S3) compared to CN IV-FN samples 4 days after subculture. The variability in VE-cadherin gene expression might stem from differences in differentiation efficiency across independent experiments, which is a common feature of stem cell differentiation due to multiple factors including batch-to-batch variability in the reagents used, some of which are animal-derived [63]. iBMECs on LN 411-E8 also exhibited similar levels of *CDH5* expression compared to CN IV-FN (see Additional file 1: Figure S8A). Finally, PECAM-1 (*PECAM1*) gene expression remained unchanged on LN 511-E8 (see Additional file 1: Figure S3) and LN 411-E8 (see Additional file 1: Figure S8A) compared to CN IV-FN.

### Subculture on LN 511-E8 impacts junctional expression of tight junction proteins

Gene expression analysis of the cells seeded on CN IV-FN and LN 511-E8 indicated no statistically significant difference in expression of ZO-1 (*TJP1*), claudin-5 (*CLDN5*), or occludin (*OCLN*) between the two groups 4 days after subculture (see Additional file 1: Figure S3). Similar results were observed between iBMECs on CN IV-FN and LN 411-E8 (see Additional file 1: Figure S8A). Immunocytochemistry of claudin-5 in iBMECs cultured on cell culture inserts shows substantial heterogeneity for the cells cultured on CN IV-FN, with areas with low expression (yellow-outlined regions in Fig. 3a) and areas with higher expression that contained mostly internalized claudin-5 (white-outlined regions in Fig. 3a). In contrast, iBMECs cultured on LN 511-E8 show more homogeneous junctional expression of claudin-5 throughout the iBMEC layer (Fig. 3b), with fewer areas of low junctional expression compared to iBMECs on CN IV-FN. Higher magnification images of claudin-5 staining on CN IV-FN from a region with low claudin-5 expression (Fig. 3c) confirms significantly weaker junctional expression compared to low claudin-5 regions on LN 511-E8 (Fig. 3d). The aforementioned heterogeneity in claudin-5 staining is not observed in the low magnification images of ZO-1 staining from the same region of iBMECs on CN IV-FN (Fig. 3e). iBMECs on LN 511-E8 also exhibit strong junctional expression of ZO-1 throughout the monolayer (Fig. 3f). However, higher magnification images of ZO-1 show a jagged pattern of ZO-1 expression in iBMECs on CN IV-FN, while iBMECs on LN 511-E8 exhibit strong and uniform junctional expression of ZO-1 (Fig. 3g, h).

Confocal microscopy of tight junction proteins 4 days after subculture on ECM protein-coated surfaces (on ibidi μ-slides) showed a substantial difference between the iBMECs cultured on CN IV-FN compared to LN 511-E8 (Fig. 3i). Claudin-5 staining was internalized, and its junctional localization was substantially reduced in most of the iBMEC layer on CN IV-FN. Moreover, occludin and ZO-1 staining demonstrated a higher number of frayed or jagged junctions (indicated by white arrows) on CN IV-FN. iBMECs on LN 511-E8, on the other hand, showed more uniform junctional expression of tight junction proteins. Similar results were obtained for iBMECs differentiated from the ACS-1024 hiPSC line (see Additional file 1: Figure S4A–H). Together, these results explain the considerably lower TEER value for the iBMECs that are cultured on CN IV-FN compared to laminin 511 on day 4 and beyond, since tight junction proteins are the key regulators of TEER.

### Stress fibers are substantially reduced in iBMECs on LN 511-E8

F-actin staining was performed in order to analyze the organization of actin filaments in iBMECs cultured on different ECM proteins. Junctional association of F-actin was higher 2 days after seeding for the iBMECs on LN 511-E8 compared to CN IV-FN (see Additional file 1: Figure S5A–D). Circumferential actin filaments were strongly expressed on LN 511-E8, while there were no visible stress fibers formed in the cells (see Additional file 1: Figure S5B, D). In contrast, formation of stress fibers was observed in iBMECs on CN IV-FN 2 days after subculture (see Additional file 1: Figure S5A, C). Furthermore, side views of the 3-D images of F-actin staining suggest a higher junctional height on LN 511-E8, which was determined by calculating the average height of the stained area (see Additional file 1: Figure S5E, F). F-actin staining 4 days after subculture showed an increase in the number of stress fibers in iBMECs on CN IV-FN, while stress fibers were still not detected in most of the iBMEC monolayer on LN 511-E8 (see Additional file 1: Figure S4I, J and S5G, H).

### Altered cell morphology observed in iBMECs on LN 511-E8

**Fig. 4:**
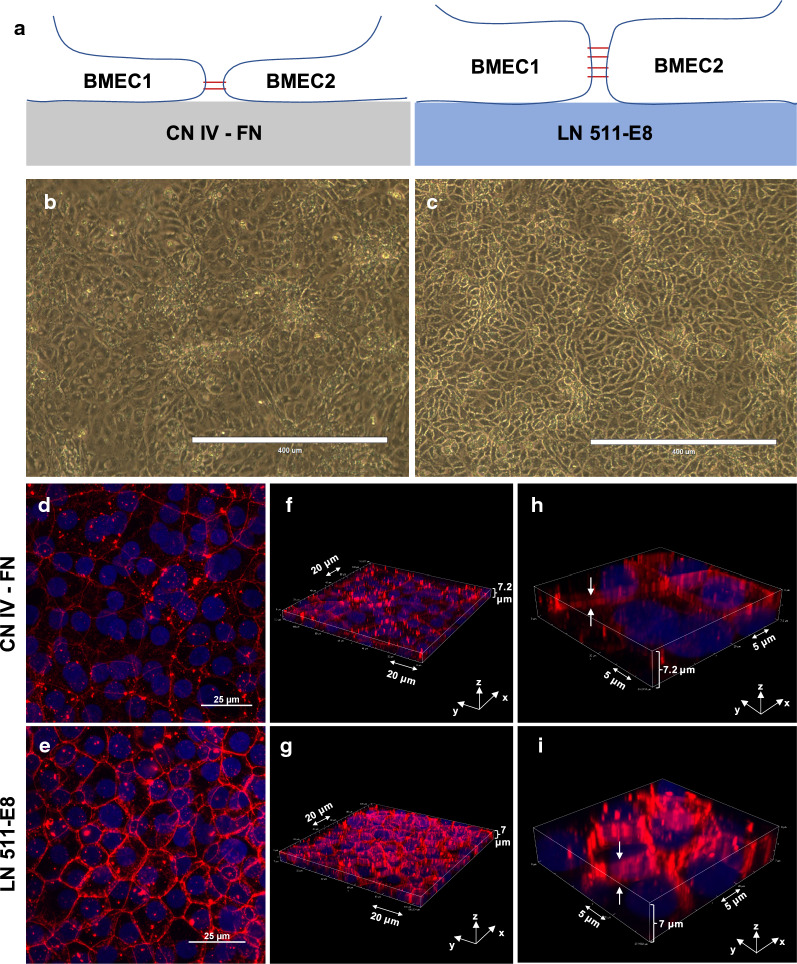
Differences in endothelial cell morphology on CN IV-FN and LN511-E8. **a** Schematic representation of iBMEC morphology on CN IV-FN (left) and LN 511-E8 (right). **b**, **c** Phase contrast images of iBMECs cultured on CN IV-FN (**b**) or LN511-E8 (**c**) 4 days after subculture. Scale bars indicate 400 μm. **d**, **e** Maximum intensity projections of confocal z-stack images of claudin-5 staining in cell culture inserts 4 days after subculture on CN IV-FN (**d**) or LN 511-E8 (**e**). **f**, **i** Three-dimensional representations of the z-stack images showing the difference in junction height on CN IV-FN (**f**, **h**) compared to LN 511-E8 (**g**, **i**)

Appearance of iBMECs on LN 511-E8 differed from iBMECs seeded on CN IV-FN. The junctions between adjacent cells were more distinct and visible on LN511-E8 (Fig. 4b, c), which might be due to the increased cell–cell contact area and more flattened cell morphology (Fig. 4a). The other noticeable difference was the formation of dome-like structures in multiple regions of the iBMEC monolayer on CN IV-FN (see Additional file 1: Figure S6A, B). These structures were not observed for cells cultured on LN 511-E8 (see Additional file 1: Figure S6C, D). These structures have been reported for the culture of epithelial cells on plates [64, 65], with detachment of cell layer from the surface due to an accumulation of fluid between the culture plate and the cells causing this phenomenon. One possible explanation for the absence of these structures on the LN 511-E8 coated plates is stronger integrin interactions with LN 511-E8, which prevent detachment of the cell layer from the ECM-coated surface. In addition, confocal z-stack and 3-D images of claudin-5 staining showed that the cell–cell junction height on LN 511-E8 was significantly higher than on CN IV-FN (Fig. 4f–i), in agreement with the results for F-actin staining (see Additional file 1: Figure S5E, F). Figure 4d and e show 2-D maximum intensity projections of the claudin-5 staining demonstrating that junctional expression was stronger on LN 511-E8. Overall, these results suggest flattened morphology with increased junctional contact between adjacent cells on the LN 511-E8 matrix, as demonstrated in Fig. 4a.

### iBMECs on LN 511-E8 demonstrate reduced fluid endocytosis rate

Confocal microscopy of iBMECs that were incubated with rhodamine B-labeled dextran indicated a lower fluid endocytosis rate in iBMECs that were cultured on LN 511-E8 compared to CN IV–FN, as evidenced by a decrease in the number of internalized fluorescent vesicles as well as lower pixel intensity of fluorescent dextran per analyzed image on LN 511-E8 (see Additional file 1: Figure S7). Low pinocytotic activity of BMECs is a distinct feature of the BBB [1], suggesting that iBMEC culture on LN 511-E8 better reflects key aspects of in vivo BBB transport mechanisms.

### iBMECs on CN IV-FN show an activated endothelial phenotype

**Fig. 5:**
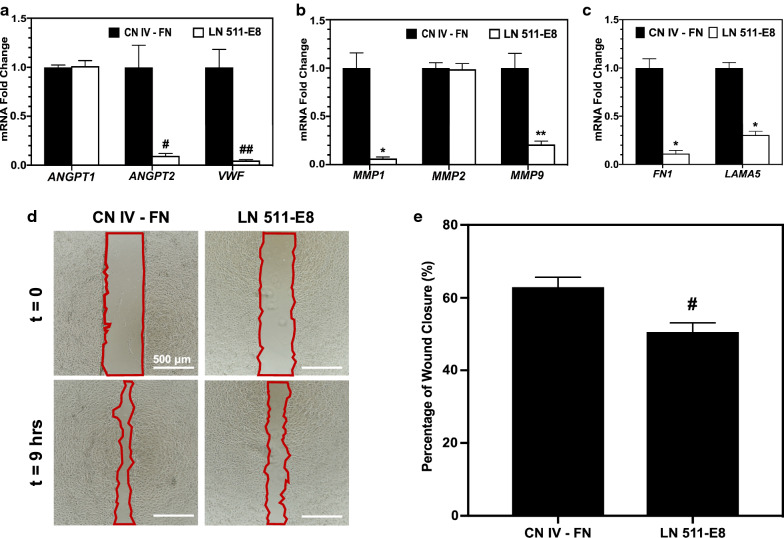
iBMECs exhibit a more activated phenotype on CN IV-FN compared to LN 511-E8. **a**–**c** Fold change in gene expression level for *ANGPT1*, *ANGPT2*, and *VWF* (**a**), various MMPs (**b**), and ECM proteins (**c**) for iBMECs on LN 511-E8 relative to iBMECs on CN IV-FN. * indicates P < 0.0005, ** indicates P < 0.001, # indicates P < 0.05, and ## indicates P < 0.01 via unpaired t-test compared to CN IV-FN for each gene. **d**, **e** Wound healing assay to assess migration of iBMECs on LN 511-E8 and CN IV-FN. **d** Representative images of the cells immediately after and 9 h after the ibidi wound healing culture inserts were removed from the plates. Cell-free area is outlined in red. Scale bars indicate 500 μm. **e** Quantification of percentage of the initial wound area that has closed after 9 h for each condition. # indicates P < 0.05 compared to CN IV-FN

Quantitative RT-PCR was conducted to quantify gene expression of angiopoietins, vWF, MMPs, and ECM proteins in iBMECs cultured on CN IV-FN, LN 511-E8, or LN 411-E8 for 4 days. As shown in Fig. 5a, *ANGPT2* and *VWF* expression was significantly higher in iBMECs on CN IV-FN compared to LN 511-E8, but *ANGPT1* expression was unchanged. *MMP1* and *MMP9* expression was also significantly higher on CN IV-FN, but the difference in *MMP2* expression was not statistically significant (Fig. 5b). In addition, expression of both *FN1* and *LAMA5* was highly upregulated in the iBMECs cultured on CN IV-FN compared to LN 511-E8 (Fig. 5c). All of the aforementioned genes, apart from fibronectin, exhibited the same pattern of differential expression on LN 411-E8 compared to CN IV-FN, but to a lesser extent (see Additional file 1: Figure S8B, C). These results were confirmed for iBMECs differentiated from the ACS-1024 cell line (see Additional file 1: Figure S4K). Next, a wound healing assay was used to evaluate the relative migration rates of iBMECs on different ECM-coated surfaces, since endothelial activation and Ang-2 upregulation are associated with increased migration of endothelial cells [66]. The rate of wound closure was higher on CN IV-FN compared to LN 511-E8 (Fig. 5d, e). Overall, the gene expression and migration results suggest iBMECs on CN IV-FN exhibit an activated endothelial phenotype.

### iBMECs seeded on LN 511-E8 show improved shear stress response

**Fig. 6:**
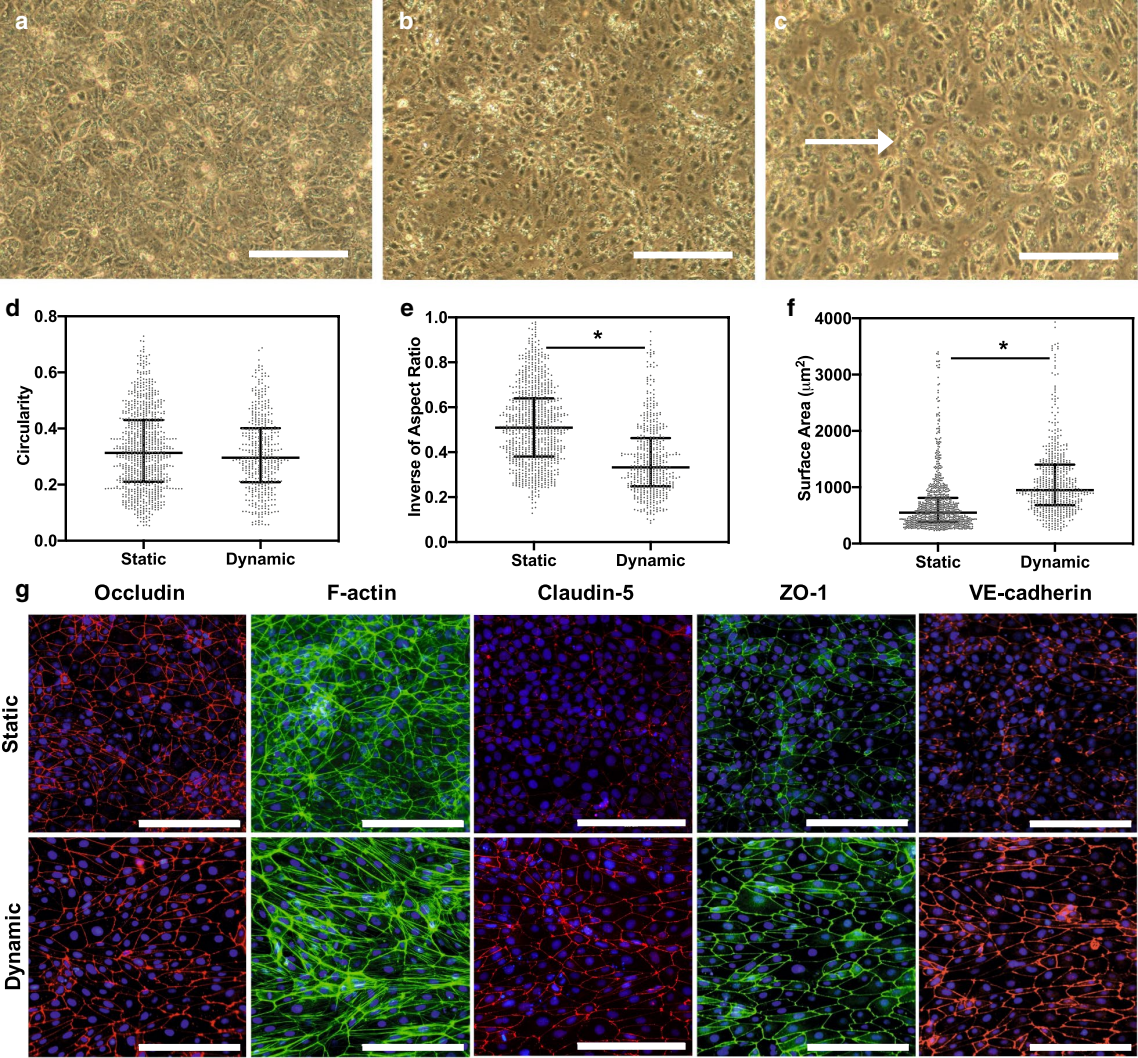
iBMECs are responsive to shear stress on LN 511-E8. **a**–**c** Phase contrast images of iBMECs before the start of flow (**a**) or under static (**b**) and dynamic (**c**) conditions for 24 h. White arrow indicates the flow direction. Scale bars indicate 200 μm. **d**–**f** Quantification of cellular shape factors including circularity (**c**), inverse of aspect ratio (**d**), average cell surface area (**e**) for iBMECs cultured under static or dynamic conditions for 24 h. Median with interquartile range are shown, and * indicates P < 0.0001 compared to static conditions. Data are representative of two biological replicates, each with 6 images analyzed per condition from three different devices. **g** Immunocytochemical analysis of iBMECs cultured for 24 h under static conditions followed by 24 h of static (top row) or dynamic (bottom row) conditions shows improvement in junctional expression of ZO-1 and VE-cadherin and the formation of stress fibers under dynamic conditions. Scale bars indicate 200 μm

Previous studies have reported a lack of expected response from iBMECs to shear stress in terms of cytoskeletal changes, elongation, and junctional protein expression [15]. However, a recent study suggested the essential role of LN 511 in the shear stress response of human umbilical artery endothelial cells and a mouse skin-derived endothelial cell line (sEND.1) through the interaction of laminin with β1 integrins [24]. Since junctional expression of PECAM-1 and VE-cadherin is required for shear stress response of endothelial cells [62, 67] and iBMECs on LN 511-E8 exhibited improved junctional expression of PECAM-1 and VE-cadherin, we hypothesized that seeding the differentiated iBMECs on LN 511-E8 might alter their response to shear stress. Morphological changes including elongation, but not alignment, of these cells and increase in average cell surface area were observed in phase contrast images of iBMECs on LN511-E8 under flow compared to static conditions (Fig. 6a–c). Using Fiji (ImageJ), the average surface area, circularity, and inverse of aspect ratio of cells under static and dynamic conditions were calculated (Fig. 6d–f). Based on the results, the inverse of aspect ratio of the cells decreased upon dynamic culture, indicating a more elongated cell morphology. Moreover, formation of stress fibers extended throughout the cytoplasm, which was absent in the static culture of these cells on LN 511-E8, was observed (Fig. 6g). Cortical F-actin in cell junctions was strongly expressed and not disrupted (Fig. 6g). Also, junctional expression of claudin-5, VE-cadherin, and ZO-1 was noticeably enhanced in iBMECs cultured under dynamic conditions (Fig. 6g). A change in occludin expression due to shear stress was not noticeable with immunocytochemistry (Fig. 6g). The effect of shear stress on iBMECs seeded on CN IV-FN was also investigated, and in agreement with a previous report [15], we did not observe a significant change in junctional protein expression (see Additional file 1: Figure S9).

## Discussion

We show here that LN 511-E8, a chemically defined fragment of a main laminin isoform of the endothelial BM, is able to improve the barrier properties of the iBMECs and alleviate the activated endothelial characteristics observed in iBMECs cultured on CN IV-FN. In the original protocol for differentiation of iBMECs and most studies that followed, CN IV-FN was used as an adhesion layer due to the ability of this blend of ECM proteins to selectively purify iBMECs from the mixture of differentiated iBMECs and neuronal cells. However, fibronectin is minimally expressed in the endothelial BM of adults [68], and the role of collagen type IV in the BM is to provide the required mechanical support [17, 22]. Laminins, on the other hand, are ubiquitously expressed in the endothelial BM of adults and are considered the biologically active components of the BM that bind to cellular integrins [17, 69]. Accordingly, in this study, we conducted the endothelial purification step on CN IV-FN and subsequently subcultured the purified iBMECs on different ECM protein-coated surfaces. LN 511-E8 was able to support long-term maintenance of a tight barrier formed by iBMECs, while there was a significant reduction in the TEER value of iBMECs on CN IV-FN after 3 or 4 days of subculture on cell culture inserts, demonstrating the suitability of using LN 511-E8 for studies that require a stable barrier over a longer period of time. Additionally, immunocytochemistry of tight junction proteins 4 days after the start of subculture demonstrated improved junctional expression on LN 511-E8 compared to CN IV-FN, specifically for claudin-5, which showed uniform junctional expression in iBMECs on LN 511-E8, but exhibited reduced or internalized patterns of expression on CN IV-FN. Claudin-5 is considered to be a gatekeeper that is a key player in maintaining BBB homeostasis, and its expression is altered in various central nervous system diseases [70]. As such, using LN 511-E8 in studies that require an intact BBB as well as in evaluating the effect of various damage inducing factors on the BBB would be advantageous.

VE-cadherin, a vascular-specific cadherin, is a key regulator of endothelial monolayer permeability. Specifically, junctional expression of VE-cadherin is crucial for maintaining vascular homeostasis [71]. However, according to published reports using iBMECs derived from similar differentiation protocols, expression of VE-cadherin is sometimes non-junctional [7] or not distinctly junctional [72] on CN IV-FN. In this study, immunocytochemical analysis of adherens junction proteins, namely VE-cadherin and PECAM-1, showed substantial enhancement in junctional association of these molecules on LN 511-E8 compared to CN IV-FN. The increased junctional expression of VE-cadherin on LN 511-E8 may also contribute to the enhanced claudin-5 expression, since junctional expression of VE-cadherin is known to affect claudin-5 expression [70, 73]. It has previously been reported that interaction of LN 511 with β1 integrins and activation of the RhoA pathway lead to strong junctional expression of VE-cadherin [16, 24]. Moreover, one possible reason for the improved VE-cadherin junctional expression on LN 511 could be internalization of VE-cadherin on CN IV- FN as a result of the interaction of endothelial cell integrin αvβ3 with fibronectin, as reported by several studies [74–76]. When we compared junctional expression of VE-cadherin on FN only, CN IV-FN, and LN 511-E8, iBMECs from the same batch of differentiation exhibited no detectable junctional expression of VE-cadherin on FN, whereas some junctional expression was observed on CN IV–FN and strong junctional association was present on LN 511-E8. VE-cadherin and PECAM-1 are present in endothelial cells throughout the body and play an essential role in maintaining BBB homeostasis, and alteration in their expression is observed in various diseases of the BBB. Thus, their improved junctional expression on LN 511-E8 is highly beneficial for in vitro BBB modeling using iBMECs.

F-actin staining of iBMECs cultured on CN IV-FN and LN 511-E8 showed a significantly higher number of stress fibers on CN IV-FN, which is indicative of an activated endothelial phenotype. As the switch from cortical actin to stress fibers is a key characteristic of the endothelial response to inflammatory stimuli, F-actin reorganization plays an important role in endothelial barrier disruption [77]. The cell culture substrate is known to be a significant factor in controlling actin filaments, and fibronectin may be a promoter of stress fiber formation [47]. Junctional association of cortical fibers in endothelial cells is accompanied by junctional maturation and changes in cell shape. As junctions mature, the area of contact between adjacent cells increases and cells become more polarized [78–80]. This could explain the higher junction height observed in 3-D immunostaining of claudin-5 and the distinct visibility of junctions on LN 511-E8 by phase contrast microscopy.

In order to further analyze the activation of iBMECs and identify the possible molecules that might be involved in destabilizing the iBMEC layer on CN IV-FN, we conducted qRT-PCR analysis, which showed upregulation of *ANGPT2*, *VWF*, *MMP1*, and *MMP9* in iBMECs cultured on CN IV-FN compared to LN 511-E8. Angiopoietins and their receptors play a critical role in angiogenesis and vascular stabilization and are almost exclusively specific to endothelial cells [81]. Angiopoietin-1 (Ang-1) is predominantly secreted by mural cells such as pericytes that wrap around mature vessels [82], whereas Ang-2 is secreted by endothelial cells and functions as an autocrine factor [53]. Angiopoietins are involved in endothelial cell–matrix interactions through binding to both ECM and the TIE-2 receptor on cells [83, 84] and upregulation of MMP-9 expression, which degrades ECM molecules [85, 86]. While Ang-1 interaction with a confluent endothelial layer is reported to enhance the barrier properties of the cell layer, Ang-2 has a destabilizing effect on endothelial cells [81, 87]. Also, Ang-2 expression is upregulated in specialized endothelial tip cells, tumor microvessels, and during hypoxic conditions [81], suggesting a key role for this molecule in activated endothelial cells. Moreover, vWF is co-localized with Ang-2 in Weibel-Palade bodies of endothelial cells and is involved in regulating Ang-2 storage and release [88]. Since Ang-2 and vWF are considered functionally related, their expression might be co-regulated [89]. MMPs are also involved in numerous disease conditions of BBB and can degrade both tight junction and ECM proteins [51, 52]. They are also active players in both physiological and pathological angiogenesis and are upregulated in activated endothelial cells [90]. Further, a wound healing assay confirmed the higher migration rate of iBMECs on CN IV-FN, which might be partly due to higher MMP activity in these cells. Additionally, mRNA levels of ECM proteins, fibronectin and laminin α5, were significantly higher on CN IV-FN. Upregulation of fibronectin and laminin α5 occurs in various diseases and during hypoxia and inflammation [36, 91]. Overall, these results show that the iBMECs on LN 511-E8 demonstrate a resting endothelial phenotype, while the iBMECs on CN IV-FN exhibit an activated phenotype.

Finally, physiological fluid flow, which is capable of inducing BBB phenotype, suppressing inflammatory signaling, and improving the tightness of the BMEC barrier [11], was added to the system to determine whether culture on LN 511-E8 could improve the ability of iBMECs to demonstrate physiological responsiveness to shear stress. Other studies have reported iBMECs do not show morphological changes or alterations in junctional protein expression due to shear stress on CN IV-FN [15, 92, 93]. In contrast, we showed that applying shear stress to iBMECs cultured on LN 511-E8, which strongly interacts with β1 integrins, can result in morphological changes and enhanced junctional association of VE-cadherin, claudin-5, and ZO-1. This is expected due to the improved junctional expression of PECAM-1 and VE-cadherin observed on LN 511-E8, as these two proteins are essential for inducing shear stress response in endothelial cells [62]. Overall, based on our results, adding a constant shear stress to iBMECs on LN 511-E8 improves their functionality by increasing the junctional expression of claudin-5, ZO-1 and VE-cadherin. This could be highly beneficial for BBB-on-a-chip platforms that add fluid flow to iBMEC cultures in order to better recapitulate the in vivo BBB by including the physiological shear stress response in vitro. In addition, since in many BBB-on-a-chip platforms iBMECs are co-cultured with astrocytes and pericytes, inclusion of LN 211, which is the main component of the parenchymal BM and has been shown to be crucial for pericyte differentiation and astrocytic endfeet polarization [94, 95], may provide further improvement in BBB function in co-culture models.

## Conclusion

In summary, the use of LN 511-E8, a fragment of physiologically relevant BM protein, improves the barrier properties and long-term stability of iBMEC monolayers and results in a resting endothelial phenotype. This is critical for enabling in vitro iBMEC models to better mimic physiological BBB conditions for studies of drug delivery or molecular transport, mechanistic studies of diseases and damage inducing factors, and investigating cancer and immune cell interactions with the BBB. The fluid shear stress response of iBMECs is also enhanced on LN 511-E8 compared to CN IV-FN. Therefore, the use of LN 511-E8 to mimic the in vivo BM significantly improves the functionality of in vitro platforms using iBMECs to model the BBB.

## Supplementary information

**Additional file 1: Table S1.** Primary antibodies used for immunocytochemistry. **Table S2.** Secondary antibodies used for immunocytochemistry. **Table S3.** Primers used for qRT-PCR assays. **Figure S1.** TEER measurement results for iBMECs differentiated from the ACS-1024 cell line. **Figure S2.** Impact of fibronectin on junctional expression of VE-cadherin. **Figure S3.** Gene expression analysis of tight junction and adherens junction proteins 4 days after subculture on CN IV-FN or LN 511-E8. **Figure S4.** Immunocytochemistry and qRT-PCR results for iBMECs differentiated from the ACS-1024 cell line 4 days after subculture on CN IV-FN or LN 511-E8. **Figure S5.** F-actin staining of iBMECs on CN IV-FN and LN 511-E8. **Figure S6.** Formation of dome-like structures in the iBMEC monolayer on CN IV-FN. **Figure S7.** Analysis of fluid endocytosis level in iBMECs on LN 511-E8 compared to CN IV-FN. **Figure S8.** Gene expression analysis of iBMECs on LN 411-E8 compared to CN IV-FN. **Figure S9.** Effect of shear stress on junctional protein expression of iBMECs cultured on CN IV-FN. **Description of cell shape analysis procedure.** Macro code used for quantifying internalized vesicles.

## Data Availability

The datasets used and/or analyzed in the current study are available from the corresponding author upon reasonable request.

## Abbreviations

Ang: Angiopoietin
BBB: Blood–brain barrier
bFGF: Basic fibroblast growth factor
BM: Basement membrane
BMEC: Brain-specific microvascular endothelial cell
CN IV: Type IV collagen
DPBS: Dulbecco’s Phosphate-Buffered Saline
ECM: Extracellular matrix
FN: Fibronectin
hiPSC: Human induced pluripotent stem cell
iBMECs: Induced brain microvascular endothelial cells
LN: Laminin
PFA: Paraformaldehyde
TEER: Transendothelial electrical resistance
