# Supplementary material for: Chemically defined human vascular laminins for biologically relevant culture of hiPSC-derived brain microvascular endothelial cells
Source: Fluids Barriers CNS. 2020 Sep 10;17:54. doi: 10.1186/s12987-020-00215-2 (PMC7488267; doi:10.1186/s12987-020-00215-2)
Supplement: Supplementary file 1 — Additional file 1: Table S1. Primary antibodies used for immunocytochemistry. Table S2. Secondary antibodies used for immunocytochemistry. Table S3. Primers used for qRT-PCR assays. Figure S1. TEER measurement results for iBMECs differentiated from the ACS-1024 cell line. Figure S2. Impact of fibronectin on junctional expression of VE-cadherin. Figure S3. Gene expression analysis of tight junction and adherens junction proteins 4 days after subculture on CN IV-FN or LN 511-E8. Figure S4. Immunocytochemistry and qRT-PCR results for iBMECs differentiated from the ACS-1024 cell line 4 days after subculture on CN IV-FN or LN 511-E8. Figure S5. F-actin staining of iBMECs on CN IV-FN and LN 511-E8. Figure S6. Formation of dome-like structures in the iBMEC monolayer on CN IV-FN. Figure S7. Analysis of fluid endocytosis level in iBMECs on LN 511-E8 compared to CN IV-FN. Figure S8. Gene expression analysis of iBMECs on LN 411-E8 compared to CN IV-FN. Figure S9. Effect of shear stress on junctional protein expression of iBMECs cultured on CN IV-FN. Description of cell shape analysis procedure. Macro code used for quantifying internalized vesicles. [file 12987_2020_215_MOESM1_ESM.pdf]

Supplementary Information

Chemically defined human vascular laminins for biologically relevant culture of hiPSC-derived brain microvascular endothelial cells

Pedram Motallebnejad, Samira M. Azarin

Supplementary Materials:

Supplementary Tables S1-S3

Supplementary Figures S1-S9

Description of cell shape analysis procedure

Macro code used for quantifying internalized vesicles

Table S1. Primary antibodies used for immunocytochemistry.

Primary Antibody (Vendor, Clone or Product Number)	Dilution Factor
Mouse monoclonal Occludin (Invitrogen, OC-3F10)	1:200
Mouse monoclonal Claudin-5 (Invitrogen, 4C3C2)	1:100
Rabbit polyclonal PECAM-1 (Lab Vision, RB10333P)	1:50
Mouse monoclonal VE-cadherin (Santa Cruz, BV9)	1:50
Rabbit polyclonal ZO-1 (Invitrogen, 40-2200)	1:100

Table S2. Secondary antibodies used for immunocytochemistry.

Secondary Antibody Conjugate (Vendor)	Dilution Factor
Goat anti mouse Alexa Fluor 488 (ThermoFisher)	1:200
Goat anti mouse Alexa Fluor 594 (ThermoFisher)	1:200
Goat anti rabbit Alexa Fluor 488 (ThermoFisher)	1:200
Goat anti rabbit Alexa Fluor 594 (ThermoFisher)	1:200

Table S3. Primers used for qRT-PCR assays.

Gene Name	Unique ID or Forward and Reverse Sequence	Vendor
VWF	qHsaCED0043330	BioRad
CDH5	qHsaCID0016288	BioRad
OCLN	qHsaCED0038290	BioRad
TJP1	qHsaCID0018062	BioRad
CLDN5	qHsaCED0047644	BioRad
ANGPT2	qHsaCID0017615	BioRad
MMP2	qHsaCED0042560	BioRad
MMP9	qHsaCID0011597	BioRad
MMP1	qHsaCED0048106	BioRad
LAMA5	qHsaCED0044330	BioRad
FN1	qHsaCED0043611	BioRad
PECAM1	Fwd sequence: TGCCGTGGAAAGCAGATACT<br>Rev sequence: TTCCAGGGATGTGCATCTGG	BioRad
GAPDH	qHsaCED0038674	BioRad
ANGPT1	qHsaCED0045626	BioRad

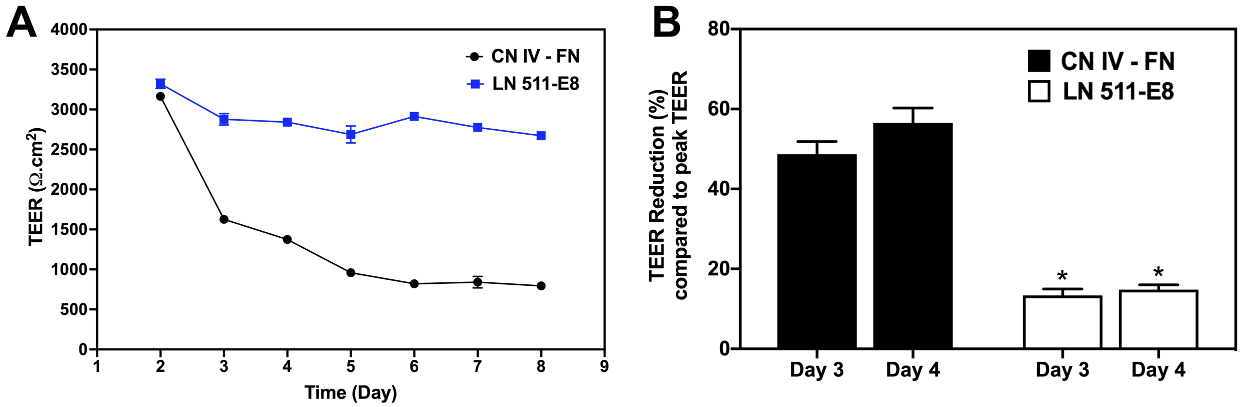

Figure S1. TEER measurement results for iBMECs differentiated from the ACS-1024 cell line. (A) TEER values for ACS-1024-derived iBMECs cultured on LN 511-E8 compared to CN IV-FN. (B) TEER reduction compared to peak TEER for ACS-1024-derived iBMECs. * indicates P < 0.005 compared to the CN IV-FN condition on the same day.

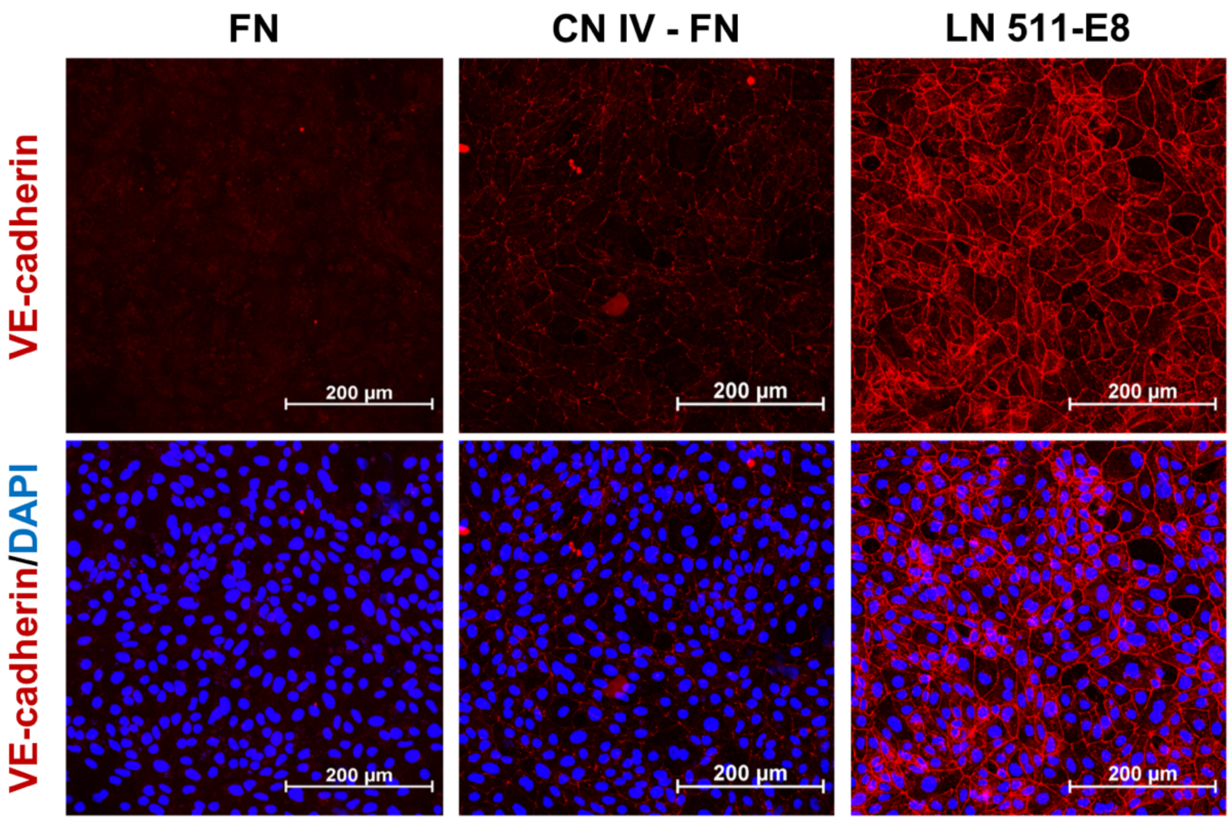

Figure S2. The presence of fibronectin decreases junctional expression of VE-cadherin. Imaging was performed 2 days following subculture of iBMECs onto the specified ECM. Top row shows images of VE-cadherin staining and bottom row shows merged images of VE-cadherin and nuclei (stained with DAPI and indicated in blue). Images are maximum intensity projections of confocal z-stacks. Scale bars indicate 200 µm.

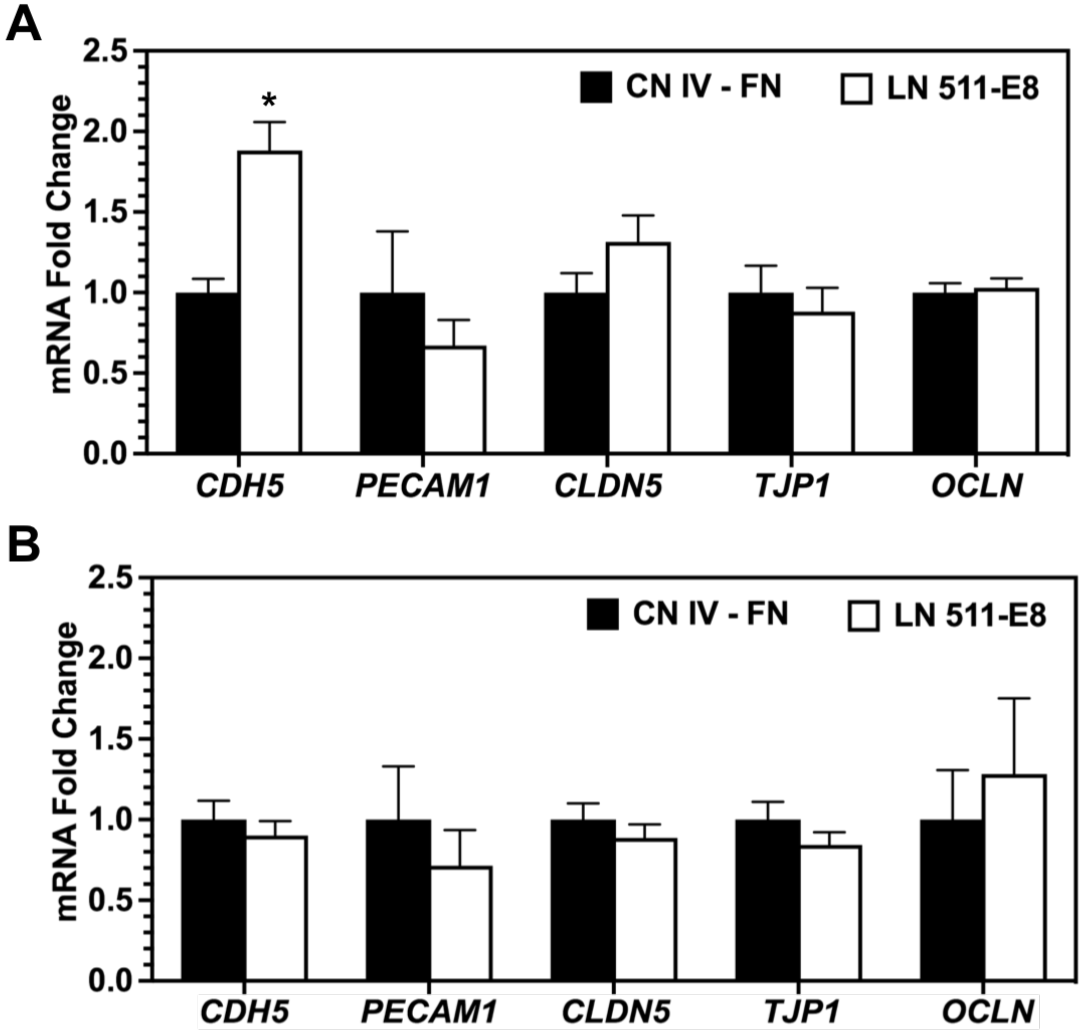

Figure S3. Gene expression analysis of tight junction (*CLDN5, TJP1, OCLN*) and adherens junction (*CDH5, PECAM1*) proteins in iBMECs on CN IV-FN or LN 511-E8 4 days after seeding on each ECM. (A) and (B) show the results for two separate biological replicates. Note that VE-cadherin (*CDH5*) expression is upregulated in (A), but unchanged in (B). Fold change is relative to CN IV-FN condition for each gene. * indicates P < 0.01 relative to CN IV-FN.

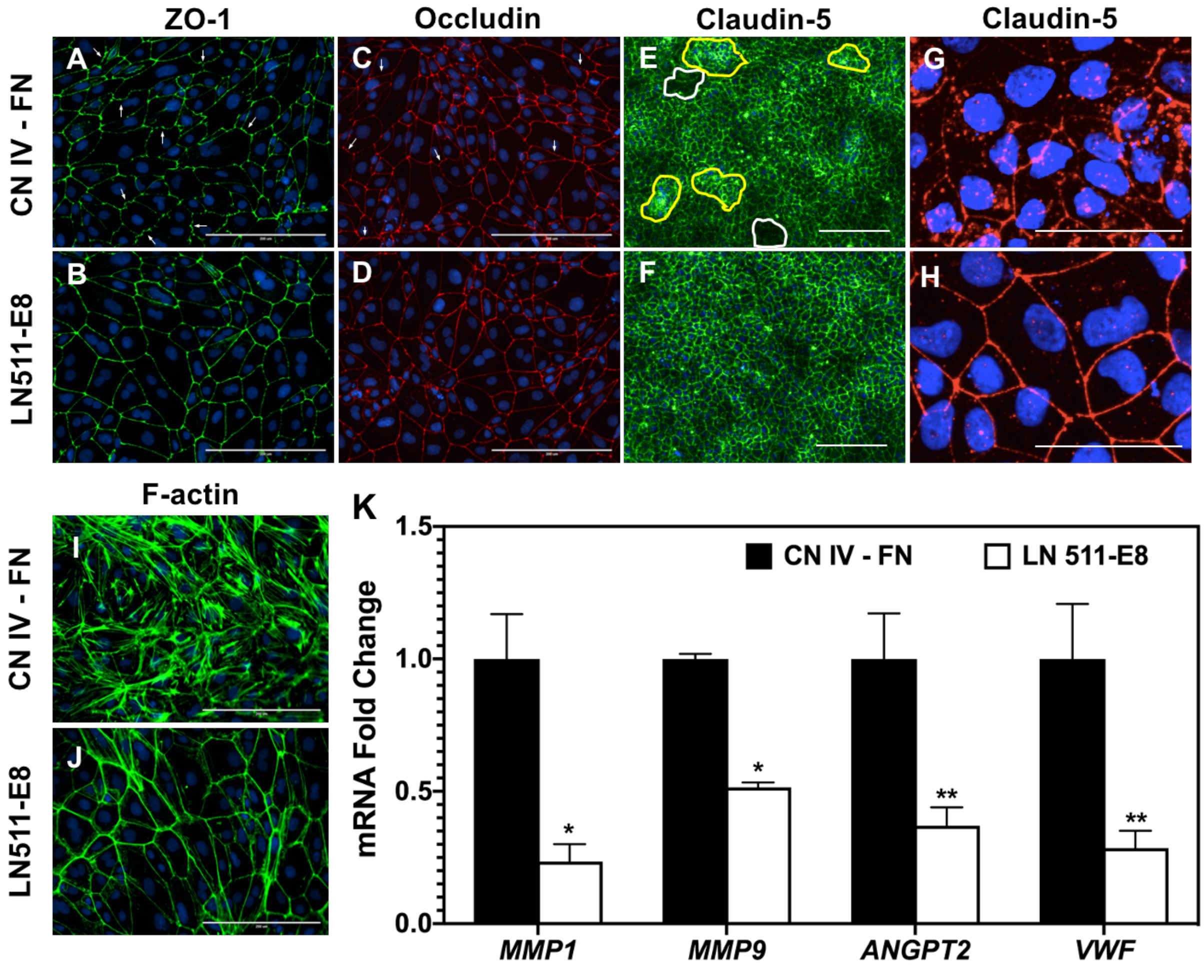

Figure S4. Immunocytochemistry and qRT-PCR results for iBMECs differentiated from the ACS-1024 cell line 4 days after subculture on CN IV-FN or LN 511-E8. (A-H) Fluorescence microscopy images of ZO-1 (A,B), occludin (C,D), and claudin-5 (E-H) on CN IV-FN (A,C,E,G) or LN 511-E8 (B,D,F,H). Scale bars indicate 200 μm for (A-F) and 25 μm for (G,H). For claudin-5 images, both low (E,F) and high (G,H) magnification images are provided. The areas outlined in yellow in (F) indicate regions with internalized claudin-5, and areas outlined in white indicate regions with low junctional claudin-5 expression. (G) and (H) show higher magnification images of regions with low claudin-5 expression. For all markers, culture on LN 511-E8 improves junctional expression. (I,J) F-actin immunostaining of iBMECs on CN IV-FN (I) or LN 511-E8 (J). Scale bars indicate 200 μm. Less stress fibers are visible in iBMECs on LN 511-E8. (K) qRT-PCR analysis of *MMP1*, *MMP9*, *ANGPT2*, and *VWF* demonstrates that expression of all four genes is significantly lower on LN 511-E8 than CN IV-FN. Fold change is relative to CN IV-FN condition for each gene. * indicates $P < 0.005$ and ** indicates $P < 0.01$ relative to CN IV-FN.

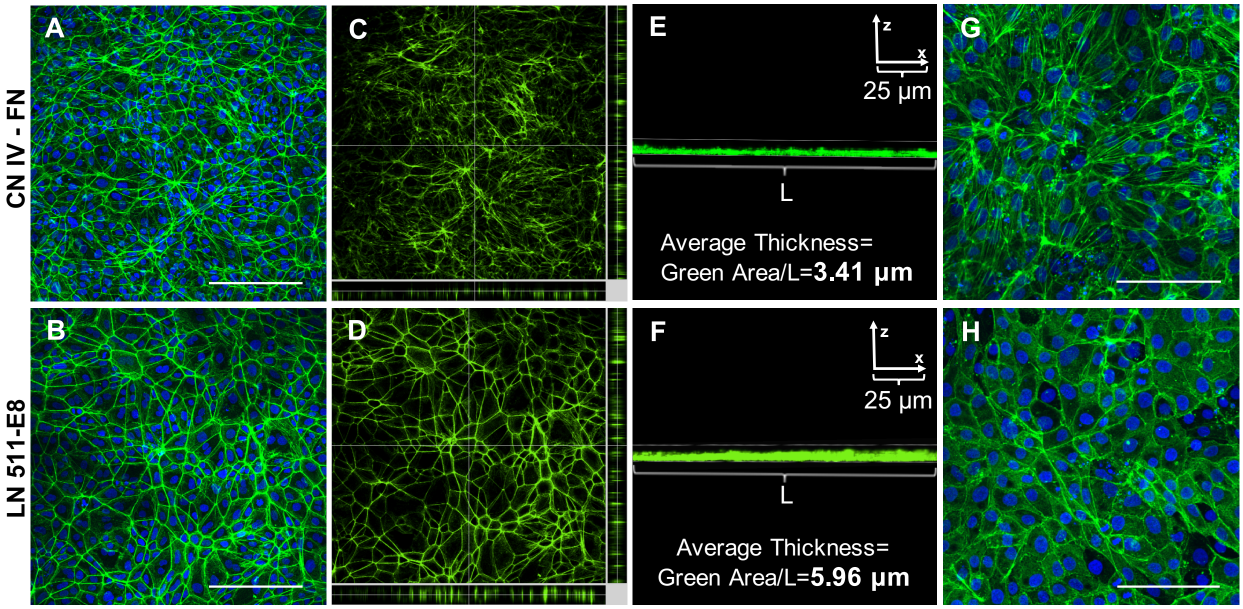

Figure S5. F-actin staining of iBMECs on CN IV-FN and LN 511-E8. (A,B) Images of F-actin (green) and nuclei (blue) for iBMECs on CN IV-FN (A) or LN 511-E8 (B) 2 days after subculture. (C,D) Middle plane image of F-actin staining with x and y axis projection of iBMECs on CN IV-FN (C) or LN 511-E8 (D) 2 days after sub-culture. Many stress fibers are visible in the CN IV-FN sample, while F-actin is mostly junctional on LN 511-E8. (E,F) Average height of F-actin expression for the iBMECs on CN IV-FN (E) and LN 511-E8 (F) 2 days after subculture. (G,H) F-actin staining 4 days after subculture on CN IV-FN (G) or LN 511-E8 (H). Nuclei are stained with DAPI and indicated in blue. Images in (A,B,G,H) are maximum intensity projections of confocal z-stacks, and scale bars indicate 100 μm.

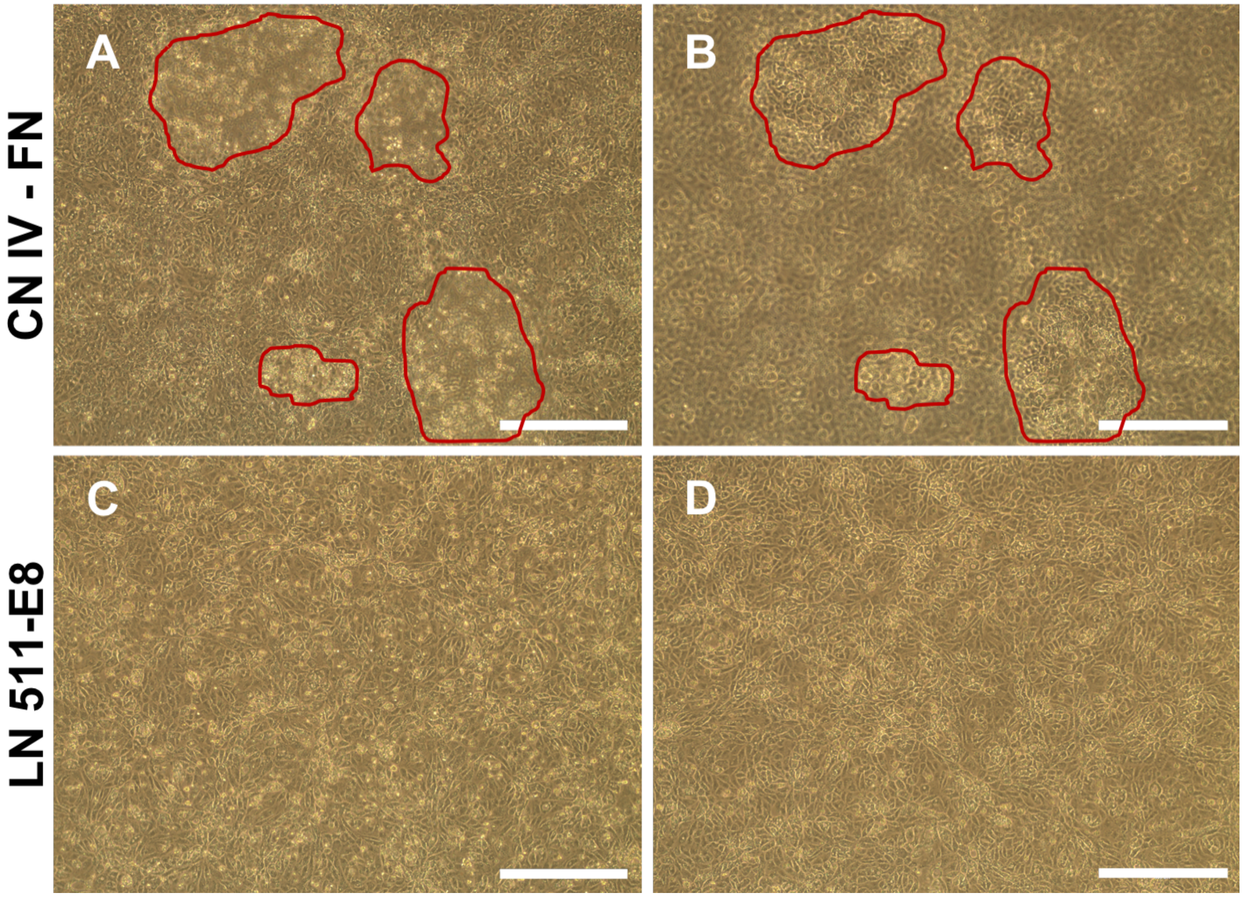

Figure S6. Formation of dome-like structures in the iBMEC monolayer on CN IV-FN. (A,B) Images from a lower focal plane (A) and upper focal plane (B) of the same region of iBMECs on CN IV-FN 4 days after subculture. Examples of dome-like structures, in which the cell monolayer is in focus at the higher focal plane, are outlined in red. (C,D) Images of two different regions of the iBMEC monolayer on LN 511-E8, demonstrating the absence of dome-like structures on LN 511-E8. Scale bars indicate 400 μm for all images.

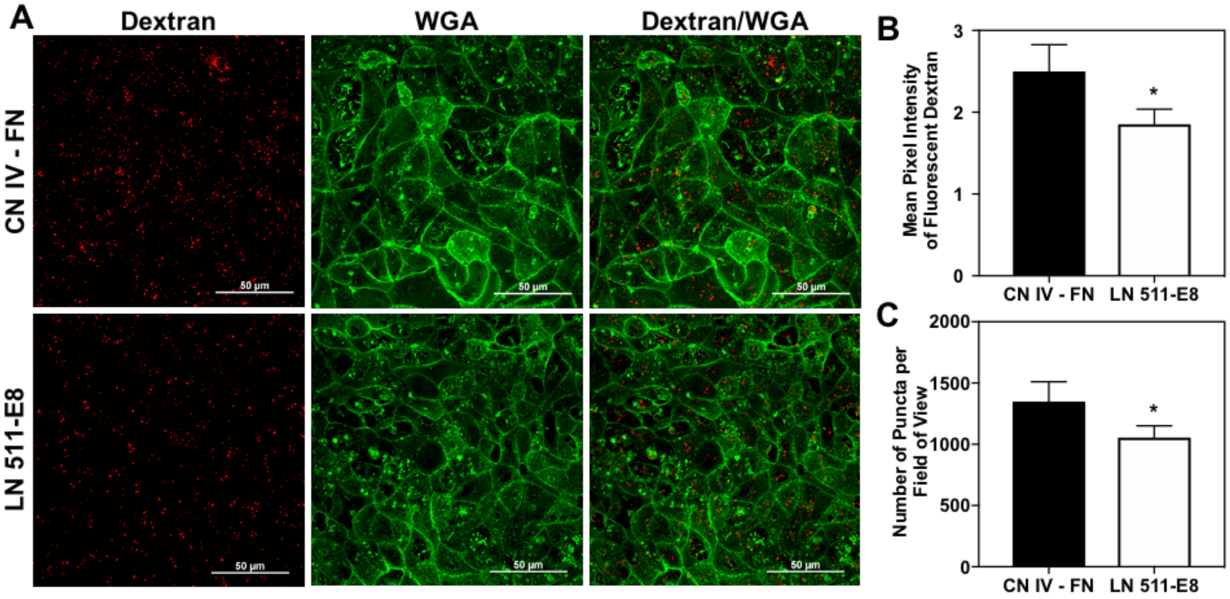

Figure S7. Analysis of fluid endocytosis level in iBMECs on LN 511-E8 compared to CN IV-FN. (A) Confocal microscopy images of iBMECs (4 days after subculture) incubated with 1 mg/ml rhodamine B-conjugated dextran, followed by fixation and staining with Oregon Green 488-conjugated WGA. Scale bars indicate 50 µm and images are maximum intensity projections of confocal z-stacks. (B,C) Quantification of mean pixel intensity (B) and the number of fluorescent puncta per image (field of view) (C) in dextran images for iBMECs on CN IV-FN and LN 511-E8. * indicates $P < 0.001$. Results represent 16 analyzed images per condition (2 images per well) from 2 independent rounds of differentiation.

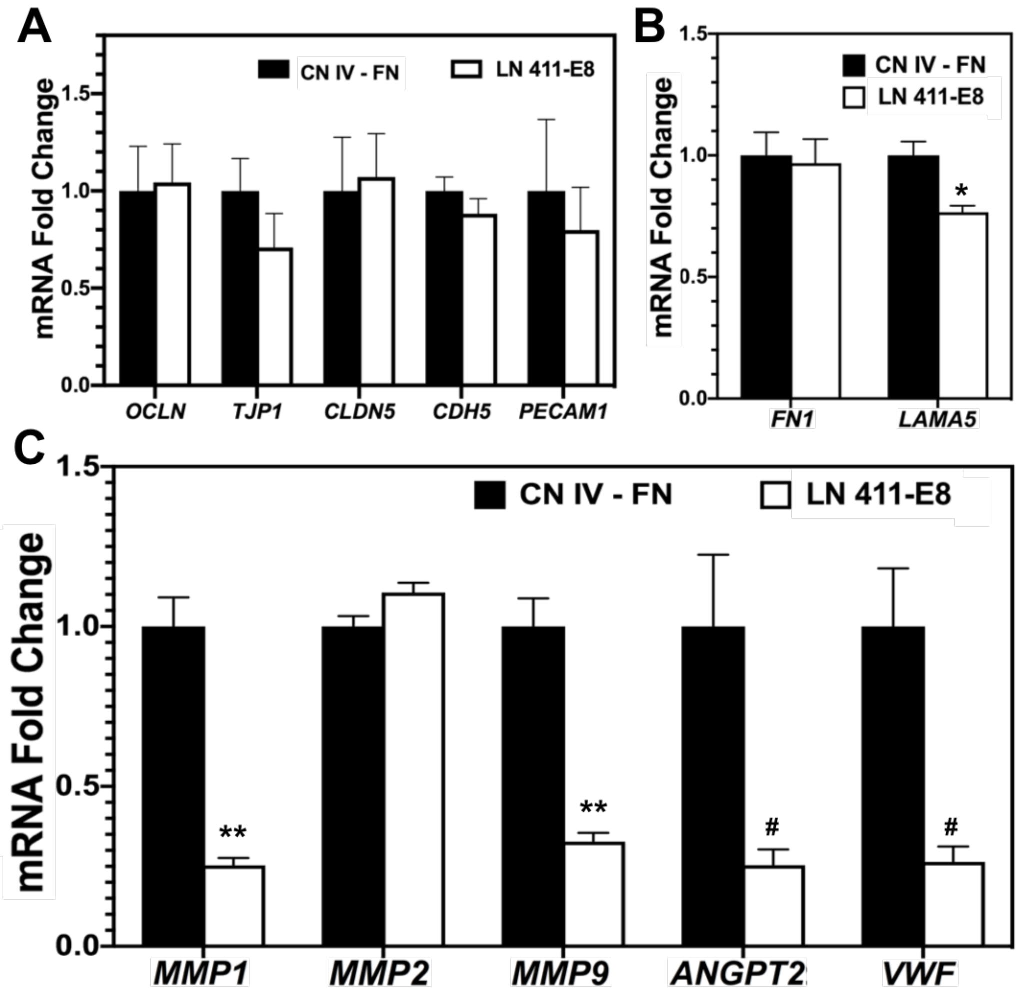

Figure S8. Gene expression analysis of iBMECs on LN 411-E8 compared to CN IV-FN. (A-C) Expression of junctional proteins (A), ECM proteins (B), and genes associated with activated endothelium (C) 4 days after subculture on each ECM. Fold change is relative to CN IV-FN condition for each gene. * indicates $P<0.05$, # indicates $P < 0.01$, and ** indicates $P < 0.005$ relative to CN IV-FN.

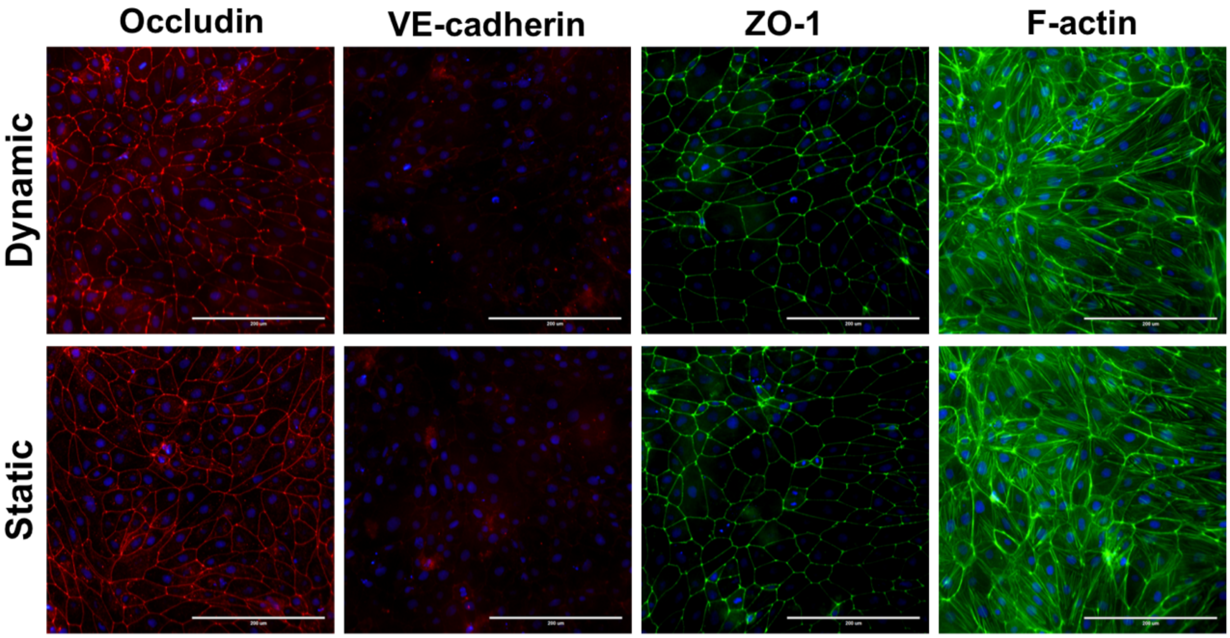

Figure S9. Effect of shear stress on junctional protein expression of iBMECs cultured on CN IV-FN. iBMECs were seeded in microfluidic devices and maintained under static conditions for 1 day to reach 100% confluency. Next, for culture under dynamic conditions, devices were connected to a syringe pump and were exposed to 4 dyne/cm^2 shear stress for 24 hours before fixation and staining of the iBMECs. Nuclei are stained with DAPI and indicated in blue. Scale bars indicate 200 µm.

Description of cell shape analysis procedure

The procedure for cell shape analysis is summarized in the macro code below. This macro can be run in Fiji (ImageJ) in order to obtain the morphological parameters of the cells in a given image. A comment (in bold font) is added to the end of each section of the code to describe the function of that part.

makeLine(1248, 910, 808, 910);
run("Set Scale...", "known=200 unit=um");
//sets scale (numbers after makeLine and run functions vary based on the image)

run("8-bit");
//converts the picture to binary

run("Subtract Background...", "rolling=20");
//reduces the noise and background

run("Find Edges");
//highlights the cell boundaries

setAutoThreshold("Huang");
setThreshold(0, 16);
run("Convert to Mask")
//adjusts the threshold and optimizes it for analysis

run("Fill Holes");
//connects the cell boundary lines

run("Analyze Particles...", "size=200-Infinity circularity=0.10-1.00 show=Outlines display exclude summarize in_situ");
//applies the shape analysis command and excludes any recognized particle with surface area smaller than 200 μm^2 and circularity smaller than 0.1 to eliminate outliers

Macro code used for quantifying internalized vesicles

The “Analyze Particle” command was used to quantify the number of vesicles per image with the macro code shown below. The number of identified particles using this code was recorded to generate the data.

```
run("8-bit");
setAutoThreshold("Default dark");
setThreshold(20, 255);
setOption("BlackBackground", false);
run("Convert to Mask");
run("Analyze Particles...", "size=2-Infinity display clear include summarize in_situ");
```
